# Incidental gallbladder lymph node metastasis from invasive ductal carcinoma

**DOI:** 10.1093/jscr/rjag1040

**Published:** 2026-11-10

**Authors:** Oghenevwoke M Onotevu, Stephaine Lau, Franz Pretorius, Georgi Atanasov

**Affiliations:** General Surgery, Goulburn Valley Health, 44-48 Graham Street, Shepparton 3630, VIC, Australia; Anatomical Pathology, Goulburn Valley Health, 44-48 Graham Street, Shepparton 3630, VIC, Australia; General Surgery, Goulburn Valley Health, 44-48 Graham Street, Shepparton 3630, VIC, Australia; General Surgery, Goulburn Valley Health, 44-48 Graham Street, Shepparton 3630, VIC, Australia

**Keywords:** breast cancer, gallbladder, cystic lymph node, metastasis, invasive ductal carcinoma, cholecystectomy

## Abstract

Breast cancer metastasis to the gallbladder or its lymphatic drainage is exceedingly rare. We report a woman in her 70s with a remote history of invasive ductal breast carcinoma who underwent elective laparoscopic cholecystectomy for symptomatic cholelithiasis. Histopathology demonstrated chronic cholecystitis and, unexpectedly, metastatic adenocarcinoma within the cystic lymph node of Lund, without involvement of the gallbladder wall or mucosa. Immunohistochemistry showed ER, PR, GATA3, and CK7 positivity with HER2 and CK20 negativity, supporting breast origin and concordance with her previous carcinoma. Subsequent staging identified axillary nodal recurrence without initial visceral or osseous metastases. Endocrine therapy with anastrozole was commenced following multidisciplinary discussion. The patient subsequently developed progressive visceral metastatic disease and died under palliative care. This case highlights the importance of histopathological assessment of cholecystectomy specimens and awareness of atypical metastatic patterns in patients with previous malignancy.

## Background

Breast cancer commonly metastasis to the bone, lung, liver, and brain [1, 2]. Involvement of the gallbladder or its lymphatic drainage is exceptionally rare and is usually reported as isolated cases or small series [3–6]. Such disease may be discovered incidentally during cholecystectomy undertaken for presumed benign biliary pathology. The cystic lymph node of Lund lies in the hepatocystic triangle and drains the gallbladder and adjacent biliary structures. Metastatic involvement by an extra-hepatobiliary primary, including breast carcinoma, is highly unusual [7]. This case describes an incidental cystic lymph node metastasis from previous invasive ductal breast carcinoma and highlights the diagnostic importance of histopathology and immunohistochemistry.

## Case presentation

A woman in her 70s residing in a nursing home presented with recurrent right upper quadrant pain and nausea. She had a history of left invasive ductal breast carcinoma treated 10 years earlier with breast-conserving surgery, radiotherapy, and 5 years of adjuvant anastrozole. Examination and laboratory tests, including inflammatory markers and liver function tests, were unremarkable. Ultrasound demonstrated multiple gallstones without acute cholecystitis (Fig. 1), while computed tomography (CT) abdomen/pelvis confirmed cholelithiasis without biliary obstruction, mass, or lymphadenopathy (Fig. 2). She underwent uncomplicated elective laparoscopic cholecystectomy for symptomatic cholelithiasis.

**Figure 1:**
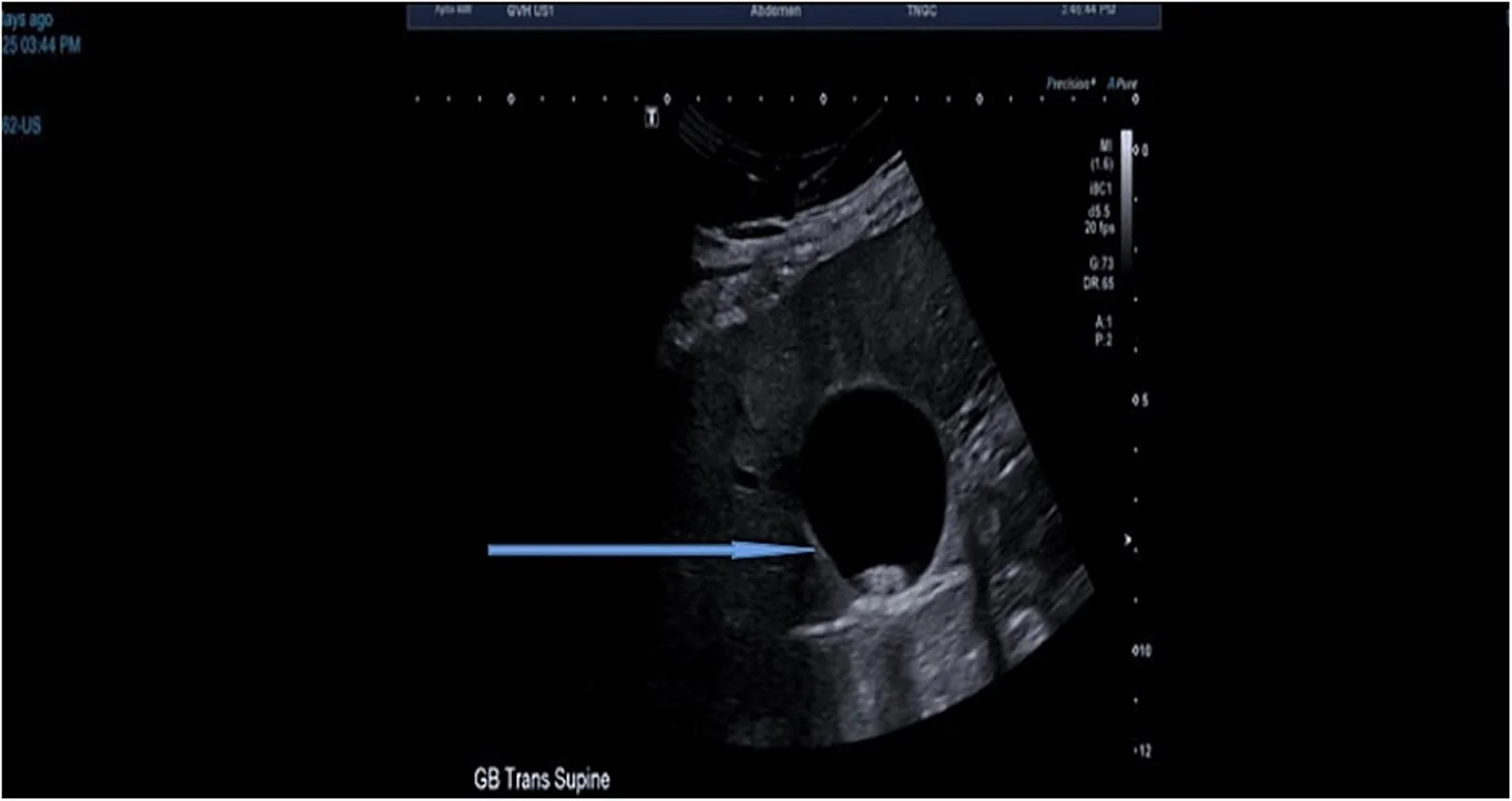
Transabdominal ultrasound of the right upper quadrant demonstrating multiple echogenic foci within the gallbladder lumen with posterior acoustic shadowing (arrow), consistent with gallstones. No gallbladder wall thickening or pericholecystic fluid is seen, in keeping with the absence of acute cholecystitis.

**Figure 2:**
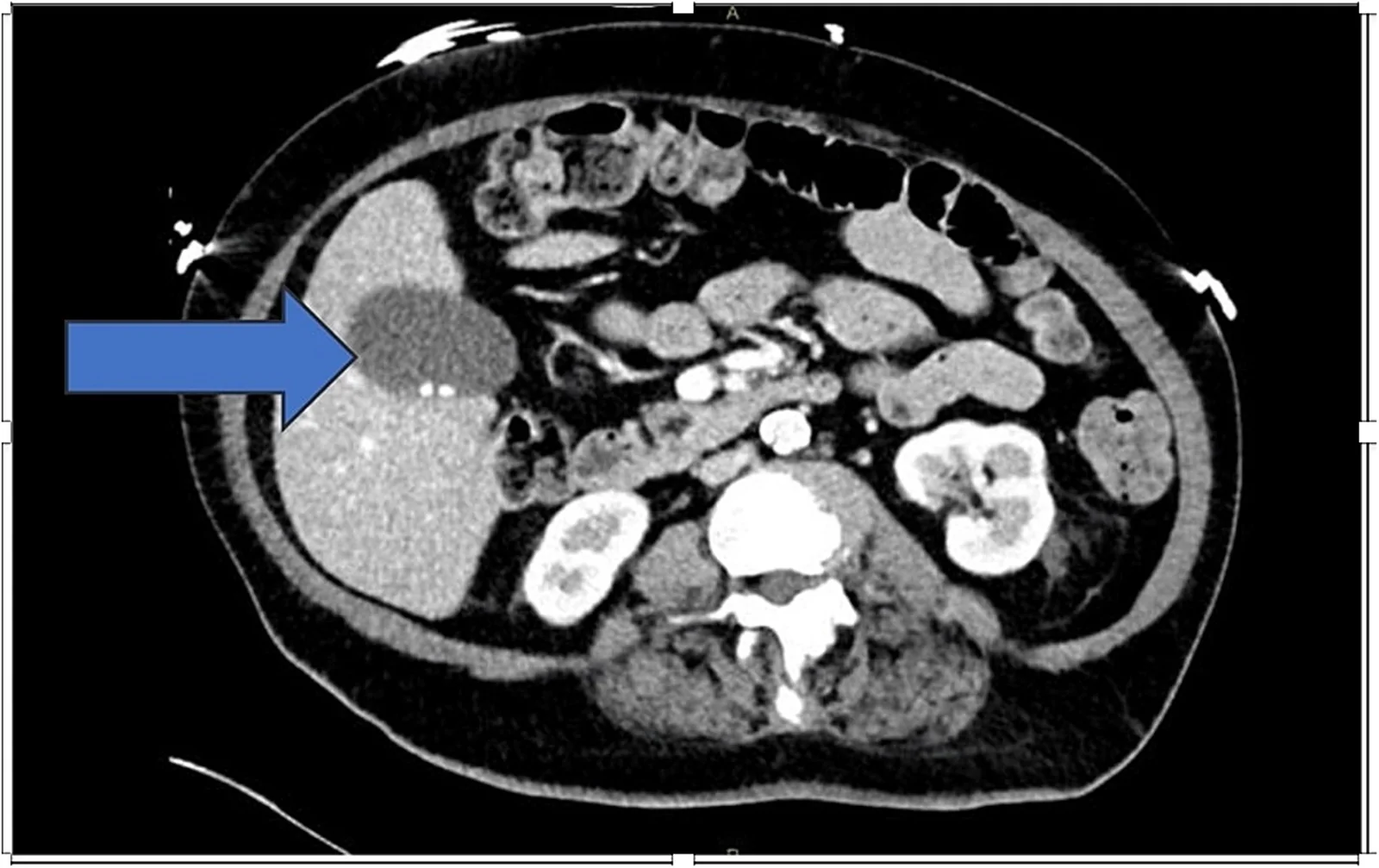
Axial portal venous phase contrast-enhanced CT of the abdomen showing multiple hyperdense calculi within the gallbladder lumen (arrow) without wall thickening, pericholecystic fluid, or biliary ductal dilatation, consistent with uncomplicated cholelithiasis.

## Investigations

Histopathology showed chronic cholecystitis with cholelithiasis and no malignancy in the gallbladder wall or mucosa. A single cystic lymph node of Lund contained metastatic adenocarcinoma. The tumor was ER-positive, PR-positive, HER2-negative, AE1/3-positive, GATA3-positive, and CK7-positive, with CK20 negativity, supporting breast origin (Figs 3–6). Breast ultrasound and mammography identified a small suspicious lesion adjacent to the previous surgical scar, although targeted biopsy was unsuccessful (Figs 7 and 8). Magnetic resonance imaging (MRI) showed stable postoperative breast changes and a benign-appearing 15 mm subareolar fibroadenoma, but a prominent left axillary lymph node with abnormal morphology was identified (BI-RADS 4A) (Fig. 9A and B). PET-CT demonstrated uptake in the left breast and axilla without visceral or osseous metastases (Fig. 10). CT brain showed no intracranial metastasis. Biopsy of the left axillary node confirmed metastatic invasive ductal carcinoma consistent with the original breast primary. Previous breast slides were reviewed alongside the new specimens and demonstrated histopathological concordance.

**Figure 3:**
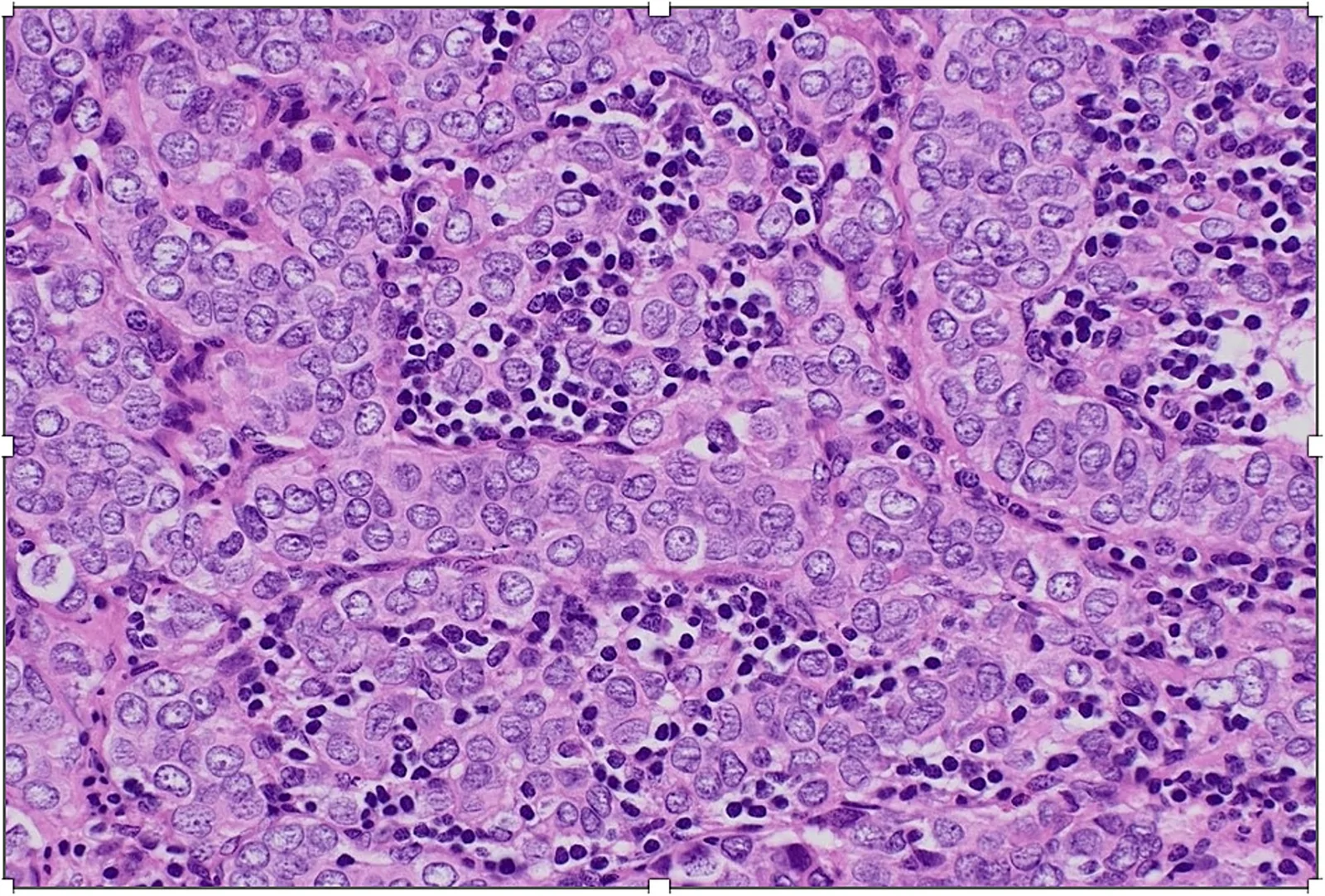
Lymph node with infiltrate of atypical epithelioid cells, showing increased nuclear to cytoplasmic ratio, irregular nuclear contours, and indistinct nucleoli (H&E stain, low power).

**Figure 4:**
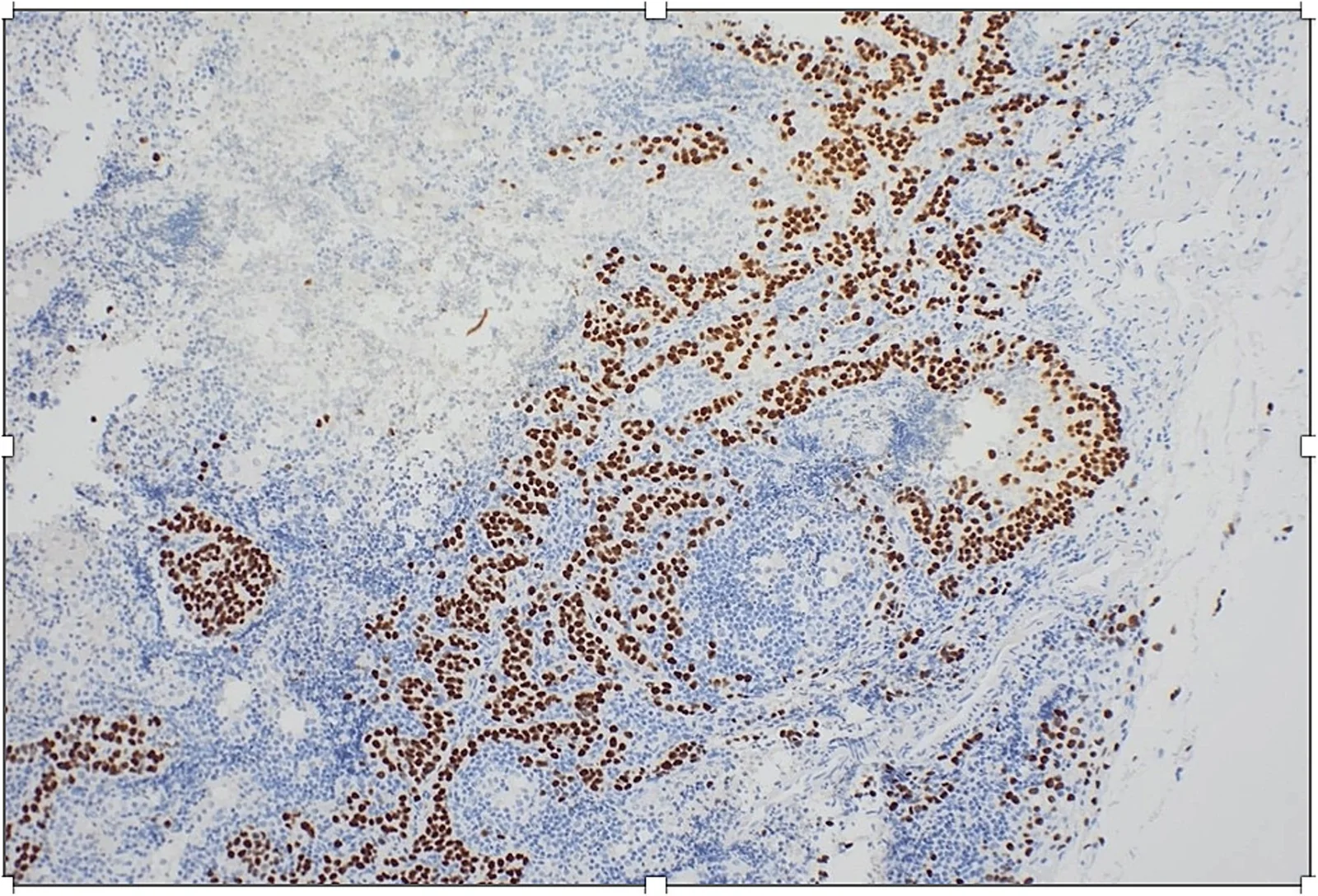
Immunohistochemistry for CK7 showing strong positivity.

**Figure 5:**
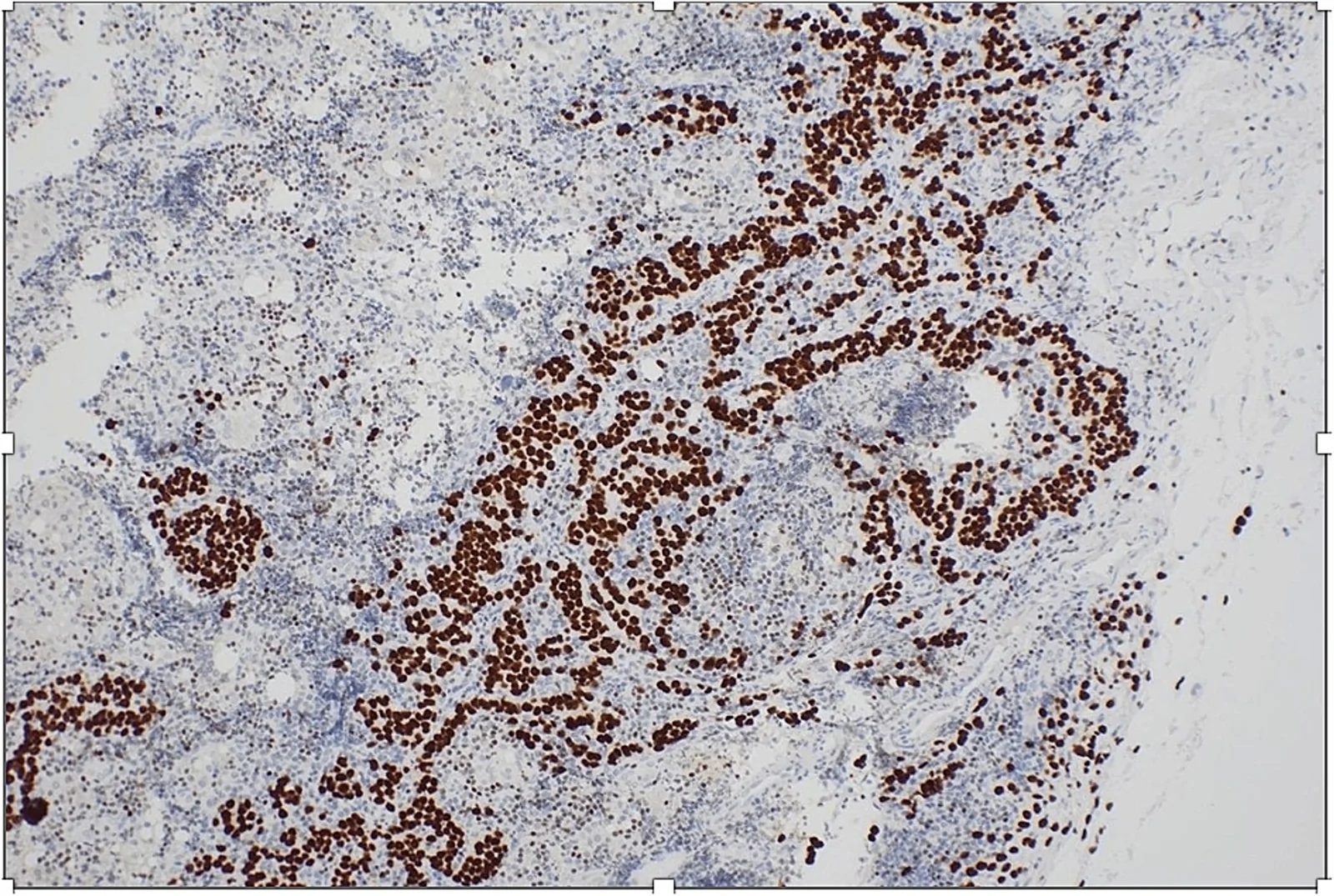
Immunohistochemistry for GATA3 showing strong positivity.

**Figure 6:**
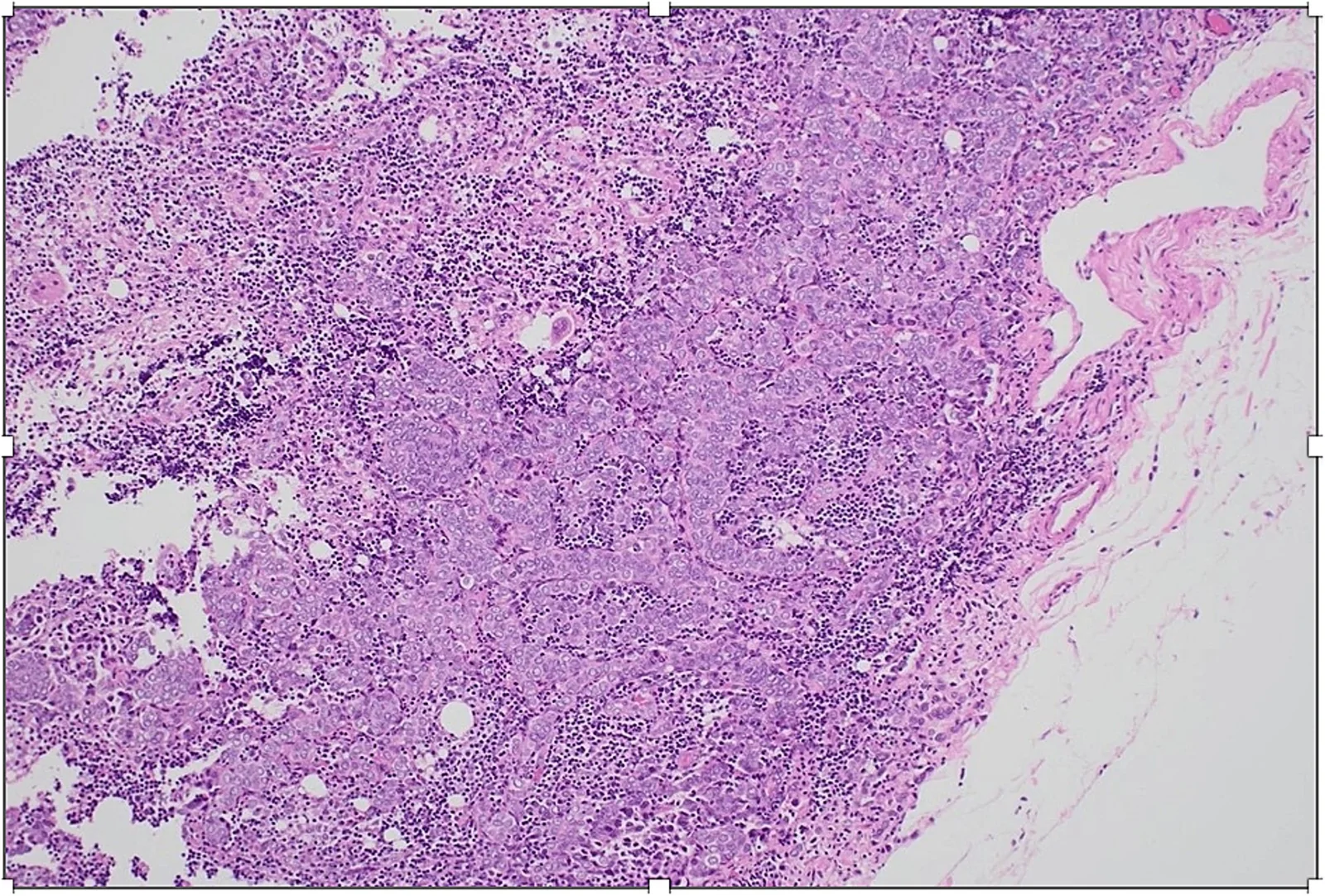
Higher magnification view demonstrating infiltrative ductal architecture of metastatic breast carcinoma (H&E stain, high power).

**Figure 7:**
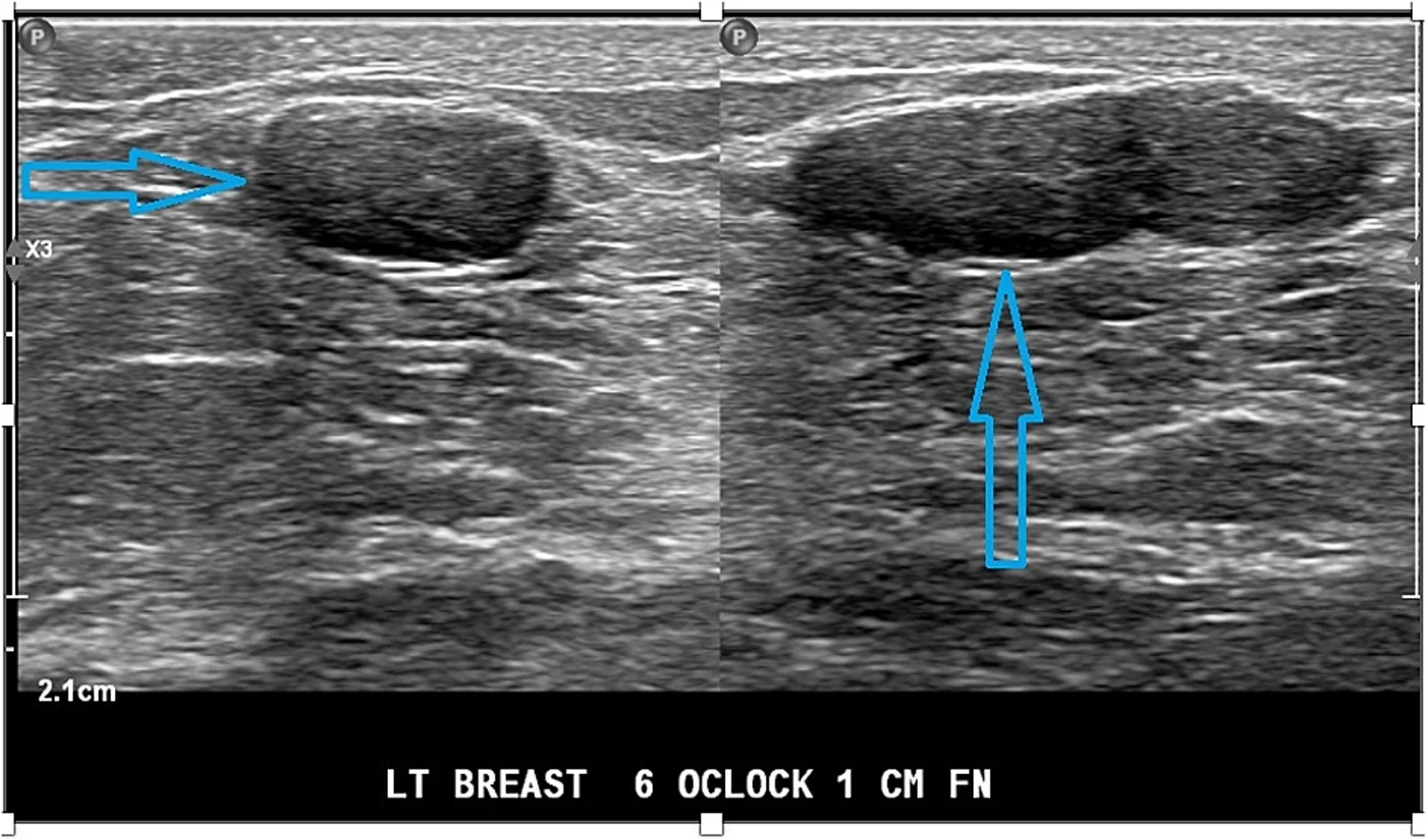
Left breast ultrasound showing a small irregular, hypoechoic, indistinct lesion at the 6 o’clock position, 1 cm from the nipple, adjacent to scar tissue, measuring 7 × 5 × 6 mm.

**Figure 8:**
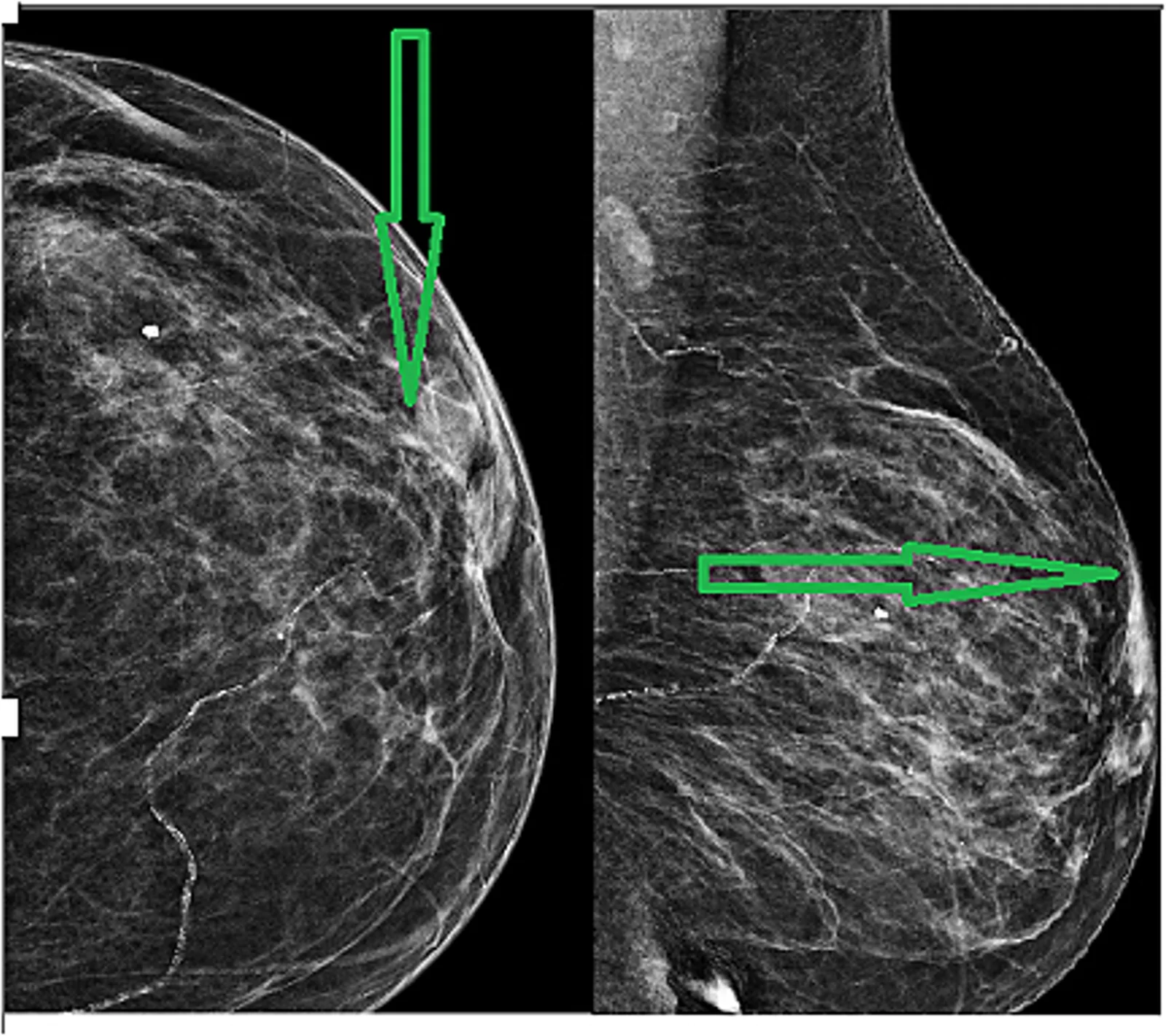
Left mammogram showing scattered areas of fibroglandular density (BI-RADS category B composition). Scarring is seen in the upper outer quadrant with a dense macrocalcification. Adjacent to the nipple is an ovoid equal-density mass with stable appearance compared with the previous mammogram. No other discrete suspicious mass or cluster of microcalcifications is identified.

**Figure 9:**
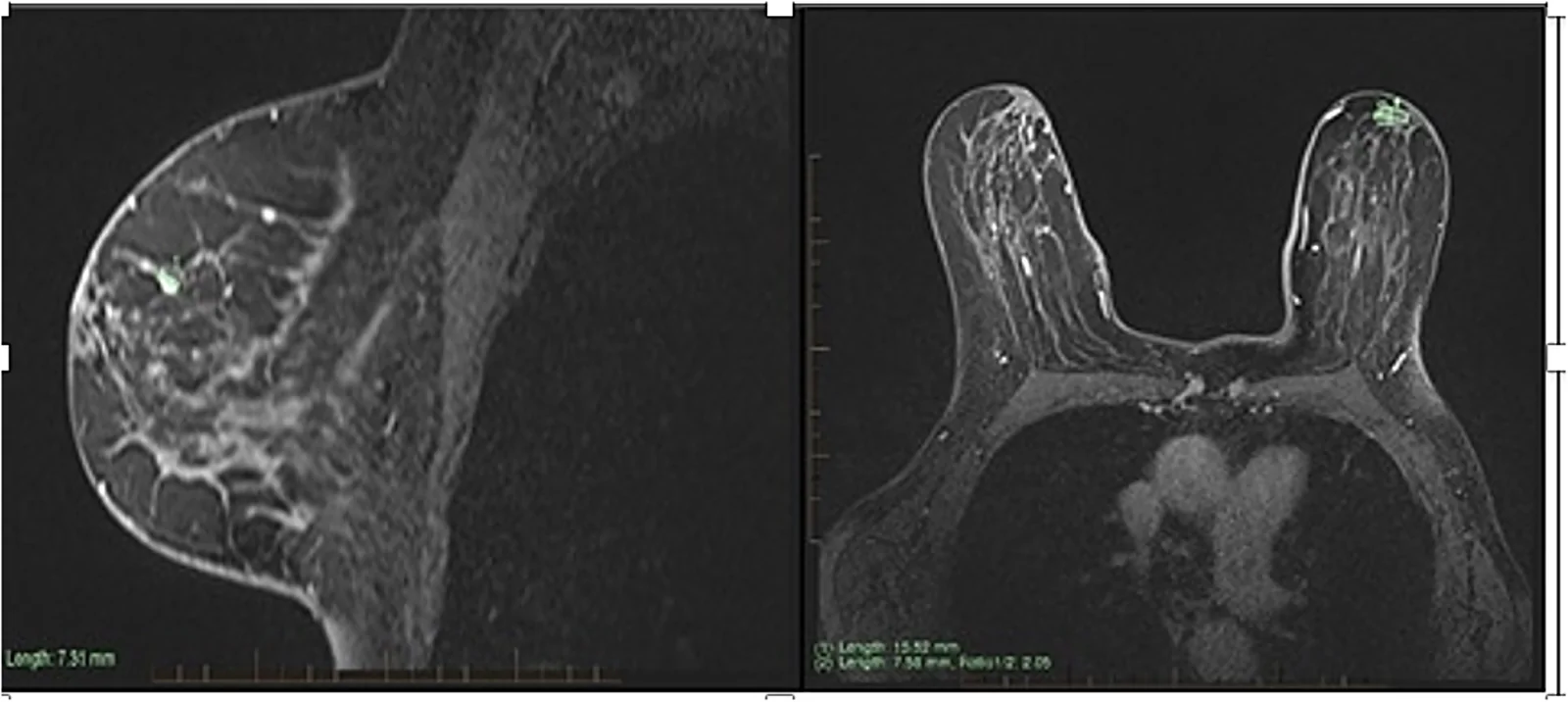
Breast MRI. (A) Axial post-contrast sequence demonstrating stable post-surgical changes and a 15 mm subareolar fibroadenoma within the left breast, without abnormal enhancement to suggest in-breast recurrence. (B) Corresponding slice demonstrating a prominent left axillary lymph node with partial loss of normal fatty hilum and reniform morphology, subsequently confirmed as metastatic on biopsy.

**Figure 10:**
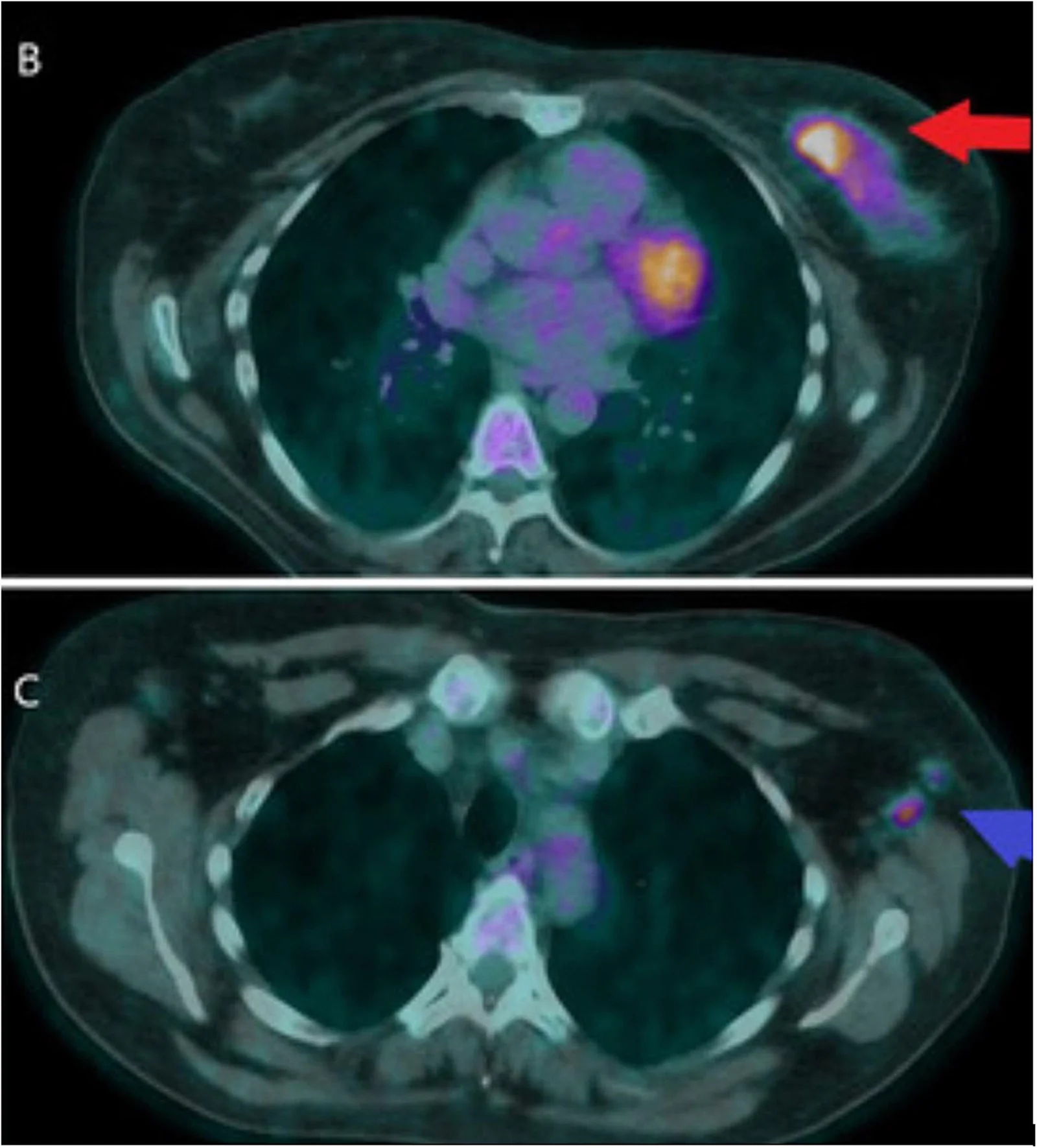
FDG PET-CT scan showing left breast and axillary uptake. No evidence of mediastinal, visceral, or osseous metastasis.

## Treatment

The patient underwent laparoscopic cholecystectomy for symptomatic cholelithiasis. Following staging and multidisciplinary review, systemic endocrine therapy with anastrozole was commenced because of hormone receptor positivity and the patient's frailty.

## Outcome and follow-up

Postoperative recovery was uncomplicated; she resumed oral intake on postoperative day 2 and was discharged on day 3. She initially remained clinically stable with acceptable tolerance of endocrine therapy, although gradual functional decline occurred in the setting of malignancy and frailty. At ~5 months, disease progression was suspected, with subsequent evidence of liver and lung metastases. Approximately 7 months after diagnosis of the cystic lymph node metastasis, her condition deteriorated and she transitioned to palliative care, where she subsequently died.

## Discussion

Breast cancer metastasis to the gallbladder or its lymphatic drainage is exceptionally uncommon and may be detected only after cholecystectomy for apparently benign disease [3–6]. The cystic node of Lund is anatomically associated with the gallbladder and biliary tract, but there is no direct lymphatic pathway from the breast to this node. Hematogenous dissemination is therefore considered a plausible mechanism [7–9]. In our patient, preoperative ultrasound and CT demonstrated uncomplicated cholelithiasis without lymphadenopathy, illustrating the difficulty of identifying microscopic nodal disease radiologically. Histopathological examination therefore provided the diagnosis and triggered further oncological staging.

Immunohistochemistry was central to establishing tumor origin. The metastatic node showed ER, PR, GATA3, and CK7 positivity with HER2 and CK20 negativity, and these findings were concordant with the patient’s previous invasive ductal breast carcinoma [10]. Although unusual gastrointestinal and peritoneal metastases are more frequently associated with invasive lobular carcinoma, invasive ductal carcinoma can also involve atypical sites, including the gallbladder and biliary tract [2, 11, 12].

The incidental nodal metastasis prompted identification of axillary recurrence and allowed systemic treatment to be considered. In a frail patient with hormone receptor-positive metastatic disease, endocrine therapy provided a less intensive treatment option following multidisciplinary discussion [11]. The subsequent development of visceral metastases and death illustrates the aggressive course that can accompany late metastatic recurrence.

This case reinforces the importance of careful histopathological assessment of cholecystectomy specimens, even when surgery is performed for presumed benign disease. It also demonstrates how an unexpected cystic lymph node metastasis can alter staging and lead to further investigation and management of an otherwise clinically occult breast cancer recurrence.

## Learning points/take-home messages

- Metastatic involvement of the gallbladder or its lymphatic drainage from breast carcinoma is exceedingly rare and may be identified incidentally after cholecystectomy.
- Histopathological examination of cholecystectomy specimens can identify clinically significant unexpected metastatic disease.
- Immunohistochemistry, interpreted with tumor morphology and clinical history, is important in establishing the origin of metastatic carcinoma.
- Management of metastatic breast cancer should be individualized according to tumor biology and patient factors.
- Awareness of atypical metastatic patterns can prompt appropriate staging and influence subsequent oncological management.
